# Protein–Protein Interactions Facilitate E4orf6-Dependent Regulation of E1B-55K SUMOylation in HAdV-C5 Infection

**DOI:** 10.3390/v14030463

**Published:** 2022-02-24

**Authors:** Marie Fiedler, Wing-Hang Ip, Helga Hofmann-Sieber, Britta Wilkens, Francis K. Nkrumah, Wenli Zhang, Anja Ehrhardt, Luca D. Bertzbach, Thomas Dobner

**Affiliations:** 1 Department of Viral Transformation, Leibniz Institute for Experimental Virology (HPI), 20251 Hamburg, Germany; marie-fiedler@web.de (M.F.); winghang.ip@leibniz-hpi.de (W.-H.I.); helgahofmann@gmx.de (H.H.-S.); britta.wilkens@leibniz-hpi.de (B.W.); francis-k-nkrumah@hotmail.de (F.K.N.); luca.bertzbach@leibniz-hpi.de (L.D.B.); 2 Center for Biomedical Education and Research (ZBAF), Department of Human Medicine, Faculty of Health, Institute of Virology and Microbiology, Witten/Herdecke University, 58453 Witten, Germany; wenli.zhang@uni-wh.de (W.Z.); anja.ehrhardt@uni-wh.de (A.E.)

**Keywords:** E3 ubiquitin ligase complex, human adenovirus, phosphorylation, post-translational modification (PTM), small ubiquitin-like modifier (SUMO), SUMO conjugation motif (SCM), SUMO conjugation site (SCS), viral replication compartment (RC)

## Abstract

The human adenovirus type C5 (HAdV-C5) E1B-55K protein is a multifunctional regulator of HAdV-C5 replication, participating in many processes required for maximal virus production. Its multifunctional properties are primarily regulated by post-translational modifications (PTMs). The most influential E1B-55K PTMs are phosphorylation at highly conserved serine and threonine residues at the C-terminus, and SUMO conjugation to lysines 104 (K104) and 101 (K101) situated in the N-terminal region of the protein, which have been shown to regulate each other. Reversible SUMO conjugation provides a molecular switch that controls key functions of the viral protein, including intracellular trafficking and viral immune evasion. Interestingly, SUMOylation at SUMO conjugation site (SCS) K104 is negatively regulated by another multifunctional HAdV-C5 protein, E4orf6, which is known to form a complex with E1B-55K. To further evaluate the role of E4orf6 in the regulation of SUMO conjugation to E1B-55K, we analyzed different virus mutants expressing E1B-55K proteins with amino acid exchanges in both SCS (K101 and K104) in the presence or absence of E4orf6. We could exclude phosphorylation as factor for E4orf6-mediated reduction of E1B-55K SUMOylation. In fact, we demonstrate that a direct interaction between E1B-55K and E4orf6 is required to reduce E1B-55K SUMOylation. Additionally, we show that an E4orf6-mediated decrease of SUMO conjugation to K101 and K104 result in impaired co-localization of E1B-55K and SUMO in viral replication compartments. These findings indicate that E4orf6 inhibits E1B-55K SUMOylation, which could favor assembly of E4orf6-dependent E3 ubiquitin ligase complexes that are known to degrade a variety of host restriction factors by proteasomal degradation and, thereby, promote viral replication.

## 1. Introduction

Adenovirus (AdV) research has greatly contributed to basic concepts of virus–host interactions and succeeded in identifying groundbreaking molecular mechanisms, such as RNA splicing or viral transformation [1,2,3]. Moreover, AdVs are extensively studied and widely used as delivery vehicles in gene therapy and vaccine applications [4]. Infections with human AdVs (HAdVs) generally cause asymptomatic or mild disease in immunocompetent individuals. In immunosuppressed patients, however, severe HAdV-induced disease is a serious health concern [5,6]. The human adenovirus type 5 of species C (HAdV-C5) is one of the most studied HAdVs and its early region 1B-55 kDa protein (E1B-55K) one of the best-characterized key players with various functions during infection [7,8,9]. Functional domains of E1B-55K include a nuclear export signal (NES) that allows CRM1-dependent and -independent nuclear-cytoplasmic shuttling [10,11,12], N-terminal SUMO conjugation sites (SCS) that facilitate SUMO conjugation to lysines at position 104 (K104) and 101 (K101) [12,13,14], a zinc finger binding domain that is known to bind viral and cellular proteins like E4orf6 [15] and p53 [16], and a C-terminal phosphorylation region (CPR) [17]. 

As a multifunctional protein, E1B-55K has been shown to, for example, prevent apoptosis [18], to counteract the host DNA damage response [9,19,20], and to efficiently promote late viral gene expression [8]. Notably, its multifunctional properties are tightly regulated, primarily through post-translational modifications (PTMs) like phosphorylation and SUMOylation [12,13,14,17,18,19,20,21]. E1B-55K localization is rather heterogeneous and dynamic and depends on cellular and/or other viral proteins and on PTMs. In general, E1B-55K diffusely localizes in the nucleus of infected cells early during HAdV-C5 infection. Later in infection, E1B-55K can be detected in viral replication compartments (RCs) and in perinuclear aggresomes [14,22,23,24,25,26].

The protein encoded by HAdV-C5 early region 4 open reading frame 6 (E4orf6) is a 34 kDa oncoprotein that promotes viral replication and oncogenic transformation [22,27]. Importantly, E1B-55K and E4orf6 form E3 ubiquitin ligase complexes that are crucial for the viral replication-promoting effect of E4orf6. These E3 ubiquitin ligase complexes catalyze the transfer of ubiquitin to cellular protein substrates including antiviral factors for subsequent proteasomal degradation [28,29,30,31,32,33]. E4orf6 has an effect on oncogenic transformation through transcription modulation and inhibition of the tumor suppressor p53 and related proteins in cooperation with E1A and E1B-55K, and, likely, also through other means in a “hit-and-run” fashion [27,34,35,36].

SUMOylation of proteins is a post-translational modification where small ubiquitin-like modifier (SUMO) proteins are covalently attached to lysine residues of target substrate proteins. SUMOylation has various molecular consequences for the modified protein [37,38]. SUMOylated HAdV proteins include the viral DNA binding protein [39], pV [40], and E1B-55K at lysine residues 101 and 104 [12,13,14,19]. E1B-55K SUMOylation requires phosphorylation [19], and conjugation of both PTMs are coordinated by cellular factors [19,20]. Moreover, there is evidence that E1B-55K SUMOylation could be regulated by E4orf6 [41]. In their work, Lethbridge et al. demonstrated that infection with E4orf6-deletion mutants led to an increase in E1B-55K SUMOylation levels [41]. However, no attempts were made to further evaluate the role of E4orf6 in E1B-55K SUMOylation regulation.

The main goal of this study was, therefore, to determine the role of E4orf6 in regulating SUMO conjugation to E1B-55K. We investigated a panel of HAdV-C5 mutants expressing E1B-55K proteins with amino acid exchanges in the SUMO conjugation sites (SCS) in the presence or absence of E4orf6 and show that protein–protein interactions are necessary for the E4orf6-dependent regulation of E1B-55K SUMOylation.

## 2. Materials and Methods

### 2.1. Cells and Culture Conditions

HeLa cells (ATCC CCL-2; American Type Culture Collection; Manassas, VA, USA), H1299 cells (ATCC-CRL-5803), and A549 cells (ACC-107; German Collection of Microorganisms and Cell Cultures; Braunschweig, Germany) were maintained in Dulbecco’s Modified Eagle Medium (DMEM; Gibco; Carlsbad, CA, USA) supplemented with 10% fetal calf serum (PAN Biotech; Aidenbach, Germany) and 1% penicillin/streptomycin solution (10,000 U/mL penicillin; 10 mg/mL streptomycin in 0.9% NaCl, PAN Biotech) at 37 °C/5% CO_2_. HeLa cells that overexpress 6×His-SUMO 1 or 6×His-SUMO 2 [42] were constantly cultivated in DMEM supplemented with puromycin (1 μg/mL) to ensure transgene expression. All cells were regularly tested for mycoplasma contamination.

### 2.2. Viruses

All virus mutants (Table 1) were generated from the HAdV-C5 wild type (wt) reference strain H5*hh*4300 by RED recombineering with primers listed in Table S1 as described earlier [43]. All introduced mutations were confirmed by Sanger sequencing.

### 2.3. Antibodies and Protein Analyses

For immunoprecipitations, total cell lysates were prepared with radioimmunoprecipitation assay (RIPA) lysis buffer (50 mM Tris/HCl pH 8, 150 mM NaCl, 5 mM EDTA, 1% P-40, 0.1% SDS, and 0.5% sodium deoxycholate), and 1 mg from each sample was precleared by Pansorbin A addition and incubation for 1 h at 4 °C on a rotator. Precleared protein lysates were mixed with antibody coupled sepharose and immunoprecipitated at 4 °C on a rotator (GFL; Lauda-Königshofen, Germany) for at least 2 h and centrifuged at 600× *g* for 5 min at 4 °C. For denaturation and protein elution, the samples were mixed with 2× SDS sample buffer (100 mM Tris/HCl pH 6.8, 4% SDS, 200 mM DTT, 0.2% bromophenol blue and 20% glycerol) and heated to 95 °C for 3 min. Finally, solubilized proteins were separated from the sepharose by centrifugation at 11,000× *g* for 3 min and analyzed by SDS-PAGE. A small fraction of each cell lysate was retained in 5× SDS sample buffer as input sample to determine steady-state protein concentrations.

For Ni-NTA SUMO pulldown analysis, we followed a published protocol for the detection of protein SUMOylation [42]. Briefly, parental HeLa cells or His-SUMO 2 cells were infected or transfected. After 24 to 48 h, cells were harvested and washed with pre-cooled PBS. Subsequently, these samples were split, whereby a small fraction, 10%, was used for the total cell lysate preparation and the remaining 90% were used for Ni-NTA SUMO pulldown analysis. Cell lysates were lysed in RIPA buffer and resolved in 5× SDS sample buffer. Ni-NTA pulldown samples were lysed in guanidine hydrochloride (GuHCl) buffer (6 M Guanidinium-HCl, 10 mM Tris and 100 mM sodium phosphate buffer pH 8.0) and His-SUMO modified proteins were coupled to the Ni-NTA agarose. Here, Ni-NTA agarose beads (Thermo Scientific; Waltham, MA, USA) were added to Ni-NTA pulldown samples and incubated overnight at 4 °C. Next, His-SUMO conjugates coupled to the Ni-NTA agarose were precipitated via centrifugation and His-SUMO conjugated proteins were eluted from the beads by addition of elution buffer (200 mM imidazole, 0.1% SDS, 150 mM Tris/HCl pH 6.3, 30% glycerol, 720 mM β-mercaptoethanol and 0.01% bromophenol blue) and heating to 95 °C for 5 min.

All samples were stored at −20 °C for further investigations by SDS-PAGE and Western blotting.

For Western blotting, SDS-PAGE separated proteins were transferred to nitrocellulose membranes that were then incubated in 5% non-fat dry milk-PBS-tween solution for 1 h at 4 °C to saturate non-specific antibody binding sites. Next, membranes were washed with PBS-tween and incubated with the respective primary antibody at 4 °C (Table 2). After 3 h of incubation, membranes were washed again and incubated with the respective HRP-conjugated secondary antibody for 2 h at 4 °C. After final washes of the membranes, proteins were visualized using the SuperSignal West Pico Chemiluminescent Substrate (Thermo Scientific; Waltham, MA, USA) according to the manufacturer’s instructions.

### 2.4. Immunofluorescence Analyses

Cells were seeded on 6-well plates with glass coverslips and infected 24 h later. Twenty-four hours post-infection (h p.i.), cells were fixed with 4% paraformaldehyde (PFA), permeabilized with PBS-Triton and blocked with TBS-BG (Tris-buffered saline with BSA and glycine) for 30 min. Next, coverslips were incubated for 1 h with indicated primary antibodies diluted in PBS (Table 2). The coverslips were washed three times with TBS-BG and indicated secondary antibodies were diluted 1/100 in PBS and added to the samples. In addition, the antibody dilution was supplemented with DAPI in a ratio of 1:5000. After a 1 h incubation and washes, coverslips were mounted on glass slides using glow mounting medium. Images were acquired with a confocal spinning-disk microscope (Nikon Eclipse Ti-E stand (Nikon; Tokyo, Japan); Yokagawa CSU-W1 spinning disk (Yokogawa; Tokyo, Japan); 2× Andor888 EM-CCD camera (Oxford Instruments, Abingdon, UK); Nikon 100× NA 1.49 objective (Nikon)), analyzed in Fiji [50] and assembled with Illustrator CS6 (Adobe; Mountain View, CA, USA). For each condition, approximately 300 infected cells were manually analyzed in Fiji and the number of cells in which both proteins co-localize was determined.

## 3. Results

### 3.1. E4orf6 Downregulates E1B-55K SUMOylation

**Figure 1:**
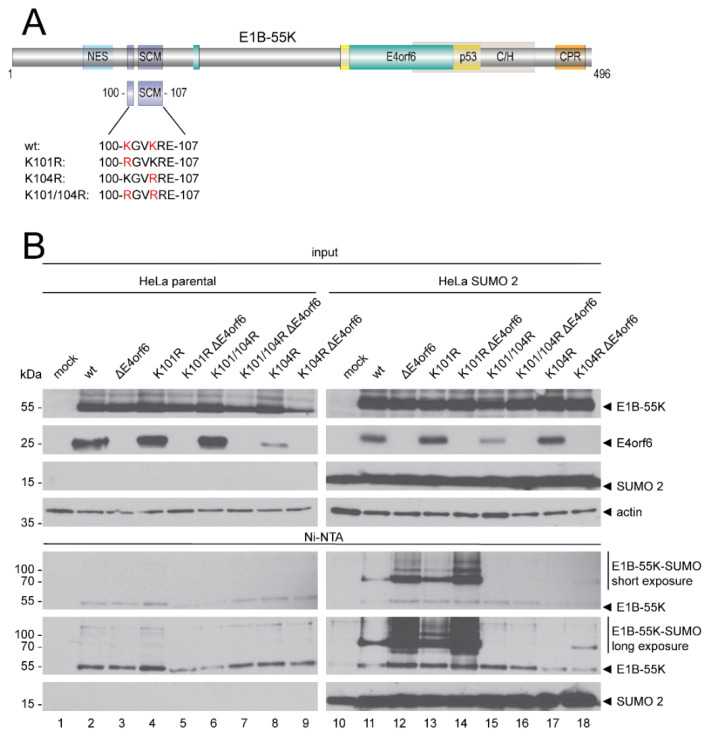
E4orf6 regulates E1B-55K SUMOylation and inhibits SUMO attachment. (**A**) Schematic overview of the HAdV-C5 E1B-55K protein. The HAdV-C5 SUMO conjugation mutants carry lysine (K) to arginine (R) substitutions at the SUMO conjugation sites (SCS) K101, K104, and K101 plus K104, respectively. NES, nuclear export signal. The turquoise E4orf6- and the yellow p53-boxes indicate the respective essential binding regions of these proteins to E1B-55K. C/H, conserved histidine (H) and cysteine (C)-rich zinc finger binding domain. CPR, C-terminal phosphorylation region. Amino acid positions are indicated. (**B**) Parental HeLa cells and HeLa cells that constitutively express His-tagged SUMO 2 were mock infected or infected with HAdV-C5 wt and indicated mutants at an MOI of 20. Cells were harvested at 24 h p.i. and His-SUMO conjugates were Ni-NTA purified. Precipitates and total cell lysates were separated according to their molecular weight by SDS-PAGE and visualized by Western blotting. For specific protein detection, mAb 2A6 (E1B-55K), mAb RSA3 (E4orf6), mAb 6×His (SUMO 1/2), and mAb AC-15 (actin) were used. Molecular weights in kDa are indicated on the left, while corresponding proteins are labeled on the right. Images represent the results of >3 repeated experiments.

E1B-55K SUMOylation is prerequisite for numerous functions of the protein and the interaction between E1B-55K and E4orf6 as its regulator is an intriguing internal viral regulatory mechanism. To investigate if E4orf6 functions as negative regulator of E1B-55K SUMOylation, we generated a set of SUMO conjugation mutants introducing amino acid exchanges in one or both SUMO conjugation sites (SCS) (K101 and K104; Figure 1A and Table 1). These mutants were used to infect parental HeLa cells and HeLa cells that constitutively express His-tagged SUMO 2, followed by Ni-NTA pulldown assays and visualization of differences in the SUMOylation levels and protein steady state concentrations by sodium dodecyl sulfate–polyacrylamide gel electrophoresis (SDS-PAGE) and Western blotting (Figure 1B).

Our data show that wt E1B-55K, as well as the K101R mutant, were modified by SUMO 2 in the presence of E4orf6, as previously reported (Figure 1B, lanes 11 and 13) [14]. In absence of E4orf6, however, we observed a much higher SUMO 2 modification of E1B-55K wt and K101R (Figure 1B, lanes 12 and 14). In contrast, K101/104R and K104R were not or very subtly modified by SUMO 2 in presence and absence of E4orf6, respectively (Figure 1B, lanes 15 to 18). Notably, we observed comparable outcomes in cells that stably express SUMO 1 (Figure S1). These results demonstrate that E4orf6 efficiently decreased SUMO conjugation to E1B-55K wt and the K101R mutant. Moreover, modest detection of SUMO conjugation upon mutation of the K104 SCM in K104R ΔE4orf6 infections confirm the newly described K101 as a functional and active SCS for the first time. Our data also once more confirm K104 as the major functional E1B-55K SCS. Conclusively, we show that E4orf6 is a negative regulator of SUMO conjugations to E1B-55K K101 and K104.

### 3.2. Specific E1B-55K SUMOylation Does Not Influence Binding to E4orf6

Next, we set to investigate if E4orf6 generally impedes protein SUMOylation or specifically reduces E1B-55K SUMO modification. We analyzed the impact of E4orf6 on SUMOylation of the viral protein E2A, another representative HAdV-C5 SUMO substrate [39]. We infected parental HeLa cells and HeLa cells overexpressing SUMO 2 with wt or ΔE4orf6 and analyzed E2A and E1B-55K SUMOylation in Ni-NTA pulldown experiments.

**Figure 2:**
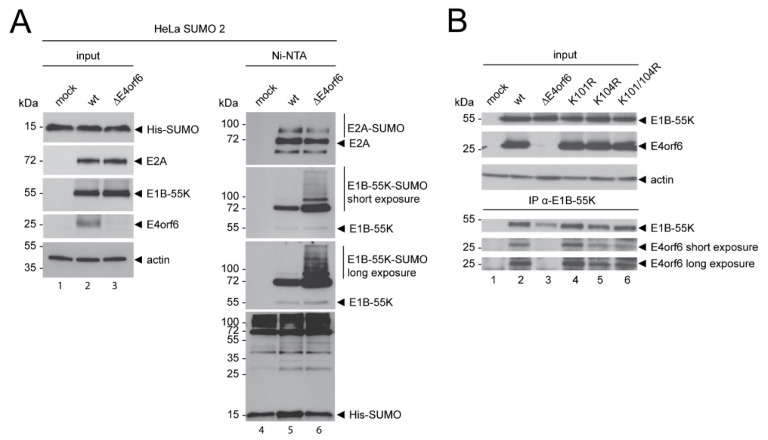
E4orf6 specifically reduces E1B-55K SUMOylation, which does not affect binding to E4orf6. (**A**) HeLa cells that constitutively express His-tagged SUMO 2 were mock infected or infected at an MOI of 20 with HAdV-C5 wt or HAdV-C5 ∆E4orf6. The cells were harvested at 24 h p.i. and His-SUMO 2 conjugates were subjected to Ni-NTA purification. (**B**) A549 cells were infected with the HAdV-C5 wt and indicated virus mutants at an MOI of 20 and harvested at 24 h p.i for an E1B-55K IP using the 2A6 mouse mAb. Total cell lysates were prepared in parallel (input). Proteins were separated by SDS-PAGE according to their molecular weight and subjected to Western blotting. Proteins were detected using mAb 2A6 (E1B-55K), mAb RSA3 (E4orf6), mAb B6-8 (E2A), mAb 6×His (SUMO 1/2), and mAb AC-15 (actin). Molecular weights in kDa are indicated on the left, while corresponding proteins are labeled on the right. Images represent the results of >3 repeated experiments.

Western blots from Ni-NTA pulldowns show that E4orf6 expression only very slightly enhances E2A SUMOylation, while E1B-55K SUMO conjugation was clearly and considerably enhanced in the absence of E4orf6 (Figure 2A). 

These observations are backed-up by comparable levels of Ni-NTA-purified high-molecular weight SUMOylated proteins (Figure 2A). Our results strongly suggest that E4orf6 does not deplete global SUMOylation, for example, by interfering with the unique E1 or E2 SUMO enzymes [51], as this would markedly decrease SUMO conjugation to all substrates, including E2A. SUMOylation can lead to altered protein–protein interactions and it is possible that E1B-55K SUMOylation influences its binding to E4orf6, as reported for other E1B-55K interaction partners [52]. To determine if E1B-55K SUMOylation affects binding to E4orf6, we immunoprecipitated E1B-55K from cells infected with different SUMO conjugation mutants and ΔE4orf6 (Figure 2B). Here, the wt and all the SCS mutants bound to E4orf6, clearly demonstrating that E1B-55K SUMOylation does not influence binding to E4orf6.

### 3.3. E4orf6 Directly Influences E1B-55K SUMOylation but Does Not Target E1B-55K Phosphorylation

**Figure 3:**
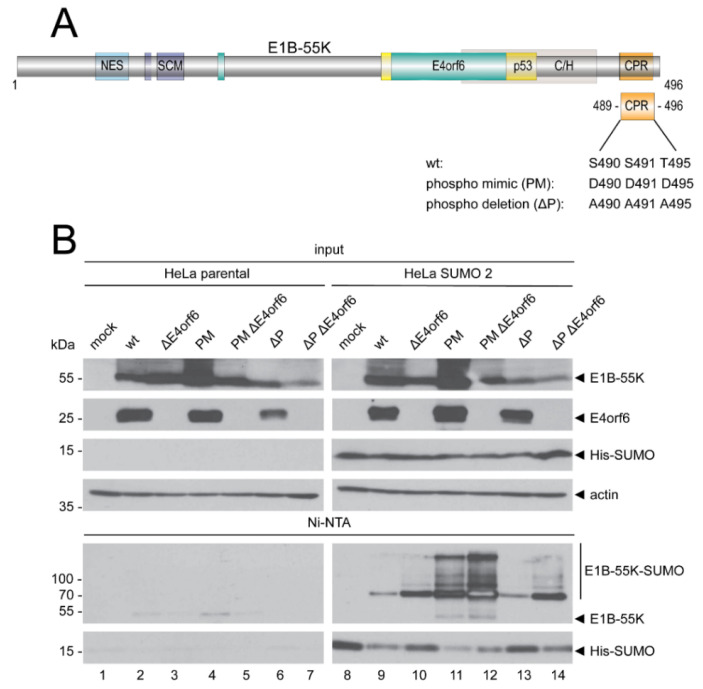
E4orf6 does not target E1B-55K phosphorylation to reduce E1B-55K SUMOylation. (**A**) Schematic overview of the HAdV-C5 E1B-55K protein. The phospho mimic (PM) and phospho deletion (ΔP) mutants with amino acid exchanges in the C-terminal phosphorylation region (CPR). Here, indicated serine (S) and threonine (T) residues are either exchanged by aspartic acid (D; PM), constantly mimicking phosphorylation, or alanine (A; ΔP), abrogating E1B-55K phosphorylation. NES, nuclear export signal. SCM, SUMO conjugation motif. The turquoise E4orf6- and the yellow p53-boxes indicate the respective essential binding regions of these proteins to E1B-55K. C/H, conserved histidine (H) and cysteine (C)-rich zinc finger binding domain. Amino acid positions are indicated. (**B**) Parental HeLa cells or HeLa cells that constitutively express His-tagged SUMO 2 were mock infected or infected with indicated viruses at an MOI of 20. Cells were harvested at 24 h p.i., total cell lysates were prepared, and His-SUMO conjugates were Ni-NTA purified. Proteins were separated by SDS-PAGE according to their molecular weight and subjected to Western blotting. Proteins were detected using mAb 2A6 (E1B-55K), mAb RSA3 (E4orf6), mAb 6×His (SUMO 1/2), and mAb AC-15 (actin). Molecular weights in kDa are indicated on the left, while corresponding proteins are labeled on the right. Images represent the results of >3 repeated experiments.

E1B-55K SUMOylation is dependent on its C-terminal phosphorylation and it has been previously shown that high E1B-55K phosphorylation levels are accompanied by high E1B-55K SUMOylation [19]. To assess a possible effect of E4orf6 on the E1B-55K phosphorylation status, which would affect the SUMO conjugation to K101 and K104, we generated HAdV phosphorylation site mutants in which the phospho-sites S490/491 and T495 were either exchanged by an alanine (A) or an aspartic acid (D) in HAdV-C5 wt and ΔE4orf6 (Figure 3A and Table 1). 

Substitutions of S490/491 and T495 with alanines prevent E1B-55K phosphorylation completely (phospho deletion, ΔP), while substitutions of the same amino acids with aspartic acids mimic a constitutive phosphorylation (phospho mimic, PM) [17,18]. Infection experiments with comparisons of ΔP and PM SUMO conjugation levels in presence and absence of E4orf6 revealed reduced SUMO 2 SUMOylation in wt and ΔP infections when compared to infections with the PM mutant, as expected (Figure 3B) [19]. The E4orf6 deletion, however, led to increased SUMOylation of the wt as well as both phospho-mutants. These data clearly demonstrate that E4orf6 does not interfere with E1B-55K phosphorylation, but directly reduces E1B-55K SUMOylation levels.

### 3.4. Interaction between E1B-55K and E4orf6 Is Required for E1B-55K SUMO Level Reductions

**Figure 4:**
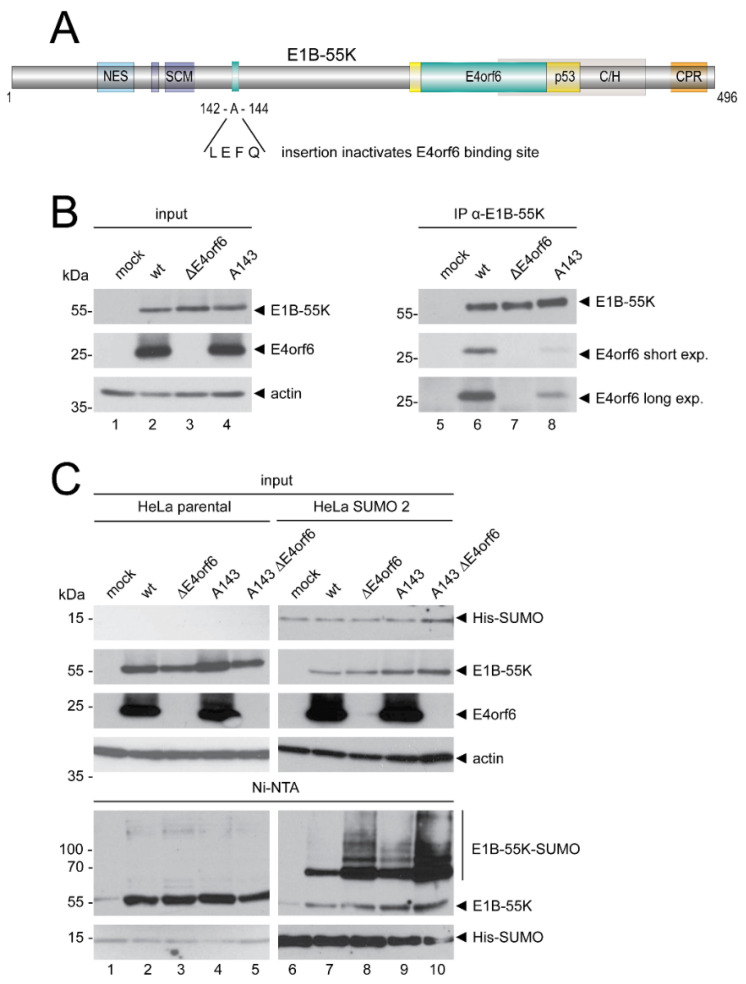
Abrogation of the interaction between E1B-55K and E4orf6 strongly increases E1B-55K SUMOylation. (**A**) Schematic overview of the HAdV-C5 E1B-55K protein. The E4orf6 binding mutant A143 with leucine (L), glutamic acid (E), phenylalanine (F), and glutamine (Q) insertions at position A143, which abrogates E4orf6 binding. NES, nuclear export signal. SCM, SUMO conjugation motif. The turquoise E4orf6- and the yellow p53-boxes indicate the respective essential binding regions of these proteins to E1B-55K. C/H, conserved histidine (H) and cysteine (C)-rich zinc finger binding domain. CPR, C-terminal phosphorylation region. Amino acid positions are indicated. (**B**) A549 cells were infected with HAdV-C5 wt and the indicated virus mutants at an MOI of 20 and harvested at 24 h p.i. Whole cell extracts were prepared for E1B-55K immunoprecipitation (IP) using the 2A6 mouse mAb. Proteins were separated by SDS-PAGE and subjected to Western blotting. (**C**) Parental HeLa cells or HeLa cells that constitutively express His-tagged SUMO 2 were mock infected or infected with indicated viruses at an MOI of 20. Cells were harvested at 24 h p.i, total cell lysates prepared, and His-SUMO conjugates were Ni-NTA purified. Proteins were separated by SDS-PAGE according to their molecular weight and subjected to Western blotting. Proteins were detected using mAb AC-15 (actin), mAb 2A6 (E1B-55K), mAb RSA3 (E4orf6), and mAb 6×His (SUMO 1/2) in Western blot analysis. Molecular weights in kDa are indicated on the left, while corresponding proteins are labeled on the right. Images represent the results of >3 repeated experiments.

E1B-55K and E4orf6 are well known to interact in order to promote viral replication. Here, we set out to investigate whether an interaction of E4orf6 with E1B-55K interferes with E1B-55K SUMOylation. We used a previously described A143 mutation harboring substitutions of four amino acids, leucine (L), glutamic acid (E), phenylalanine (F), and glutamine (Q), instead of the alanine (A) at position 143 of E1B-55K (Figure 4A) [53]. This L-E-F-Q insertion results in an E1B-55K mutant that is deficient in E4orf6 binding (Figure 4). We first verified the E4orf6 binding deficiency of the A143 mutant by E1B-55K immunoprecipitation (Figure 4B). As anticipated, precipitation of E4orf6 was significantly decreased in infections with the A143 mutant, while E1B-55K wt strongly bound to E4orf6 (Figure 4B, lanes 6 and 8). However, the interaction between A143 and E4orf6 was not completely abolished (Figure 4B, lane 8 (E4orf6 long exposure)). Next, we performed SUMO pulldown experiments from wt-, ΔE4orf6-, A143-, and A143 ΔE4orf6–infected cells. As expected, and shown before, we observed increased E1B-55K SUMOylation in ΔE4orf6-infections when compared to wt-infected cells (Figure 4C). Intriguingly, E1B-55K SUMOylation levels were higher in A143 infections when compared to wt, although they did not reach levels of ΔE4orf6 infection (Figure 4C). Concomitantly, infections with A143 ΔE4orf6 resulted in elevated E1B-55K SUMOylation levels. These findings reveal that E4orf6 and E1B-55K need to interact to reduce E1B-55K SUMOylation levels and suggest that E4orf6 might compete with SUMO as well as with the E2 or E3 SUMO enzymes for E1B-55K binding sites.

### 3.5. E1B-55K and E4orf6 Complex Formation Is a Prerequisite for Reduced SUMOylation

**Figure 5:**
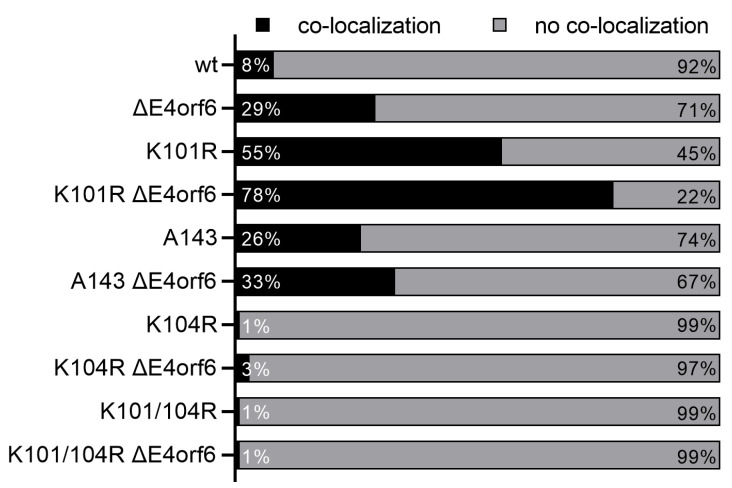
E4orf6 modulates the co-localization of E1B-55K and SUMO2 in viral RCs. Quantifications of E1B-55K and SUMO 2 co-localizations in A549 cells (Figure S2). For each virus infection, approximately 300 infected cells were analyzed and the number of cells in which both proteins co-localize was determined. The respective numbers are indicated in percentages.

To further confirm that E1B-55K and E4orf6 binding influences E1B-55K SUMOylation and additionally investigate the nature of this interaction, we analyzed localization patterns of SUMO and E1B-55K using the wt virus, the A143 mutant, and the SCS mutants by immunofluorescence. It is conceivable that E4orf6 influences co-localization of E1B-55K and SUMO 2 because it has been shown that SUMOylation of E1B-55K seems to correspond to its subcellular localization [12,13]. SUMO 2 was diffusely spread throughout the nucleus of mock-infected cells and occasionally accumulated in dots, which are presumably promyelocytic leukemia protein nuclear bodies (PML-NBs) [54]. Upon infection, SUMO 2 was re-localized either to structures resembling viral E4orf3/PML tracks or viral RCs (Figure 5 and Figure S2). Confocal microscopy and quantification of co-localizations between E1B-55K and SUMO 2 confirmed that E4orf6/E1B-55K complex formation is required for reduced SUMOylation (Figure 5 and Figure S2). We observed tremendous changes in E1B-55K and SUMO 2 co-localization patterns using the different viruses. While overlapping signals in wt infections could only be detected in 8% of all analyzed cells, the K101R mutation, which has increased E1B-55K SUMOylation levels, led to an increase of up to 55%. Infections with the same E1B-55Ks, but without E4orf6, changed the phenotype to 29% and 78%, respectively, meaning that the E4orf6 deletion resulted in increased co-localization of E1B-55K wt and the K101R mutant in viral RCs of more than 20%. Analyses of A143 infections revealed that the direct interaction between E1B-55K and E4orf6 is responsible for these changes in co-localization. Here, the binding-deficient E1B-55K mutant protein co-localized with SUMO 2 in 26% of the cells and the E4orf6 deletion only led to a modest increase (33%). These results nicely fit to the data obtained from the SUMO pulldowns in which we showed that the interaction between E1B-55K and E4orf6 is required for E1B-55K SUMO level reductions.

In contrast, the majority of E1B-55K proteins accumulated in cytoplasmic aggregates during infection with the non-SUMOylated HAdV-C5 mutants K104R, K104R ΔE4orf6, K101/104R, and K101/104R ΔE4orf6, respectively (Figure 5 and Figure S2). We found that K101/K104R was neither SUMOylated in presence nor absence of E4orf6, while K104R was slightly SUMO 2 modified in the absence of E4orf6, but not in presence of E4orf6 (Figure 1B). The lack of SUMOylation of both E1B-55K mutants resulted in a defect in viral RC localization as well as SUMO 2 co-localization and could be explained by this phenotype, since RC-association correlates with a high SUMOylation [55,56]. We occasionally observed K104R and K101/104R forming nuclear dots, which randomly co-localized with SUMO 2 (Figure S2). However, the deletion of E4orf6 did neither result in an increased nuclear accumulation of K104R and K101/104R nor in an association of K104R and K101/104R with SUMO 2 in viral RCs.

These results show that E4orf6 influences the co-localization of E1B-55K and SUMO 2 and diminishes the localization of E1B-55K to viral RCs. Furthermore, data from A143 and A143 ΔE4orf6 infections indicate that binding between E4orf6 and E1B-55K is required for the altered distribution of E1B-55K.

## 4. Discussion

The multifunctional properties of the HAdV-C5 E1B-55K protein are primarily regulated through PTMs including phosphorylation at highly conserved C-terminal serine and threonine residues and SUMO conjugation to N-terminal lysines. SUMOylation of E1B-55K is an important PTM as it regulates various functions of the oncoprotein such as SUMO ligase activity on p53 and proteins of the PML-NB complex, intracellular localization and, thus, E1A-mediated transformation of primary rodent kidney cells [12,13,14,19,52,57,58,59]. Interestingly, a report by Lethbridge and colleagues revealed that infection with E4orf6-deletion mutants led to an increase in E1B-55K SUMOylation levels [41]. To follow up on that phenomenon and to further evaluate the influence of E4orf6 on E1B-55K SUMOylation, we generated different HAdV-C5 mutants with amino acid exchanges in the E1B-55K SUMO conjugation sites, its phosphorylation sites as well as its E4orf6 binding site and investigated their SUMOylation levels in the presence or absence of E4orf6.

We first determined the E1B-55K SUMOylation status in infections with virus mutants carrying K to R exchanges of amino acid residues 101 and 104 in the presence or absence of E4orf6 and showed that E4orf6 negatively regulates E1B-55K SUMOylation by impeding SUMO attachment (Figure 1). Interestingly, we observed that the K101R SUMO levels were higher compared to wt, which was expected and has also been described recently [14]. The K101R mutation strongly affects E1B-55K SUMOylation as well as its nucleo cytoplasmic shuttling, an effect that is most probably due to regulation of the main SCS K104 [14]. Next, we investigated potential mechanisms that could be employed by E4orf6 to reduce E1B-55K SUMOylation. Investigated mechanisms included (i) inhibition of cellular SUMO-activating and -conjugating enzymes by E4orf6, (ii) E4orf6-mediated regulation of E1B-55K phosphorylation and thereby, SUMOylation [19], and (iii) a possible competition for the E1B-55K binding site between E4orf6 and SUMO proteins.

Various viral proteins reduce overall SUMOylation levels in infected cells by inferring with the SUMO conjugation machinery [60]. This has been shown for the GAM1 protein from a fowl adenovirus, for example, or human papillomavirus (HPV) E6 [61,62]. We, therefore, investigated if E4orf6 specifically reduces E1B-55K SUMOylation and verified the observation that E4orf6 deletion results in increased E1B-55K SUMOylation in H1299 cells, as HeLa cells express HPV E6 and E7 (Figure S3) [63]. Next, we compared E4orf6-dependent E1B-55K SUMOylation with SUMOylation of the HAdV-C5 protein E2A as a representative SUMO target that does not interact with E4orf6 (Figure 2) [39]. As E2A SUMOylation levels were not altered in the presence or absence of E4orf6, we concluded that E4orf6 does not interfere with SUMO-activating and -conjugating enzymes in general. Moreover, we ruled out the possibility that E1B-55K SUMOylation influences its binding to E4orf6 (Figure 2).

C-terminal phosphorylation of E1B-55K by the cellular protein kinase CK2 is a critical prerequisite for its SUMOylation [19,20]. To determine if E4orf6 targets E1B-55K phosphorylation to indirectly reduce E1B-55K SUMOylation, we tested HAdV-C5 phosphorylation mutants and revealed that neither continuous E1B-55K phosphorylation nor abrogation of E1B-55K phosphorylation altered the E4orf6-mediated deSUMOylation (Figure 3). These data clearly argue against an indirect effect via phosphorylation and imply a direct reduction of E1B-55K SUMOylation levels by E4orf6. Therefore, we used a virus mutant with a mutated E1B-55K-E4orf6 binding site to prove whether an abrogation of the interaction between E1B-55K and E4orf6 could increase E1B-55K SUMOylation. Indeed, we could show that not only the presence of E4orf6, but its direct binding to E1B-55K is necessary to reduce E1B-55K SUMOylation (Figure 4). Our observation that E1B-55K SUMOylation levels were much higher in A143 infections compared to wt, but did not reach levels of a ΔE4orf6 infection, could be explained by residual binding of A143 to E4orf6 which is sufficient enough to modestly reduce A143 SUMOylation. Nevertheless, these experiments unequivocally show that an interaction between E1B-55K and E4orf6 is required for E1B-55K SUMO level reductions. Finally, we also show that E4orf6 controls the co-localization of E1B-55K and SUMO 2 in viral RCs, virus-induced nuclear structures that promote viral replication (Figure 5 and Figure S2) [64,65]. E4orf6 diminishes the localization of E1B-55K to viral RCs, most likely to preserve its multifunctionality and prevent nuclear accumulations of E1B-55K. With these data, we demonstrate that E4orf6 decreases E1B-55K SUMOylation through protein–protein interaction. It is tempting to speculate that E4orf6 could compete with the corresponding SUMO-conjugating enzyme for binding sites on E1B-55K, again presumably to favor the assembly of the viral E3 ubiquitin ligase and enhance the nuclear export of E1B-55K to stimulate late viral gene expression [66]. Whether E4orf6 recruits SUMO-specific proteases [67] to facilitate E1B-55K deSUMOylation or not, remains to be investigated.

Taken together, we revealed E4orf6 as a negative regulator of E1B-55K SUMOylation. We showed that the E1B-55K deSUMOylation is mediated by E4orf6 through interaction between both proteins and excluded indirect effects like E1B-55K phosphorylation or global modulation of the host SUMOylation machinery. Furthermore, our findings indicate that E4orf6 inhibits E1B-55K SUMOylation to favor assembly of E4orf6-dependent E3 ubiquitin ligase complexes to degrade host restriction factors and promote an optimal environment for virus replication. These results provide important new insights into the interaction between these two regulatory multifunctional HAdV-C5 proteins and their role in the virus replication cycle.

## Figures and Tables

**Table 1:** HAdV-C5 wt and Virus Mutants.

Virus	Abbreviation	E1B-55K SUMOylation Levels Compared to wt	Reference
HAdV-C5 wt	wt	n.a.	[44]
HAdV-C5 ΔE4orf6	ΔE4orf6	higher	this study
HAdV-C5 K101R	K101R	higher	[14]
HAdV-C5 K101R ΔE4orf6	K101R ΔE4orf6	higher	this study
HAdV-C5 K104R	K104R	no SUMOylation	[12]
HAdV-C5 K104R ΔE4orf6	K104R ΔE4orf6	no SUMOylation	this study
HAdV-C5 K101/104R	K101/104R	no SUMOylation	this study
HAdV-C5 K101/104R ΔE4orf6	K101/104R ΔE4orf6	no SUMOylation	this study
HAdV-C5 E1B-55K A143	A143	higher	this study
HAdV-C5 A143 ΔE4orf6	A143 ΔE4orf6	higher	this study
HAdV-C5 ΔP	ΔP	comparable	[19]
HAdV-C5 ΔP ΔE4orf6	ΔP ΔE4orf6	higher	this study
HAdV-C5 PM	PM	higher	[19]
HAdV-C5 PM ΔE4orf6	PM ΔE4orf6	higher	this study

n.a., not applicable.

**Table 2:** Antibodies.

Antibody	Concentration	Company or Reference
Mouse mAb AC-15 (β-actin)	1:5000 (WB)	Sigma-Aldrich (St. Louis, MO, USA)
Mouse mAb 6×His (SUMO 1/2)	1:5000 (WB)	Clontech (Mountain View, CA, USA)
Mouse mAb M114-3 (SUMO 2/3)	1:100 (IF)	MoBiTec (Göttingen, Germany)
Rat mAb 4E8 (E1B-55K)	1:10 (IF)	[45]
Mouse mAb 2A6 (E1B-55K)	1:10 (WB)	[46]
Mouse mAb B6-8 (E2A)	1:10 (WB)	[47]
Mouse mAb RSA3 (E4orf6)	1:10 (WB)	[48]
Rabbit pAb 1807 (E4orf6)	1:10 (WB)	[49]
HRP α-mouse IgG	1:10,000 (WB)	Jackson (West Grove, PA, USA)
HRP α-rabbit IgG	1:10,000 (WB)	Jackson
pAb α-mouse Alexa 488	1:100 (IF)	Invitrogen (Carlsbad, CA, USA)
pAb α-rat Alexa 555	1:100 (IF)	Invitrogen

WB, Western blot; IF, immunofluorescence; mAb, monoclonal antibody; pAb, polyclonal antibody.

## Data Availability

Not applicable.

## Supplementary Materials

The following supporting information can be downloaded at https://www.mdpi.com/article/10.3390/v14030463/s1: Figure S1, E4orf6 regulates E1B-55K SUMOylation and inhibits SUMO attachment; Figure S2, E4orf6 modulates the co-localization of E1B-55K and SUMO2 in viral RCs; Figure S3, Validation of the E4orf6-dependent E1B-55K SUMOylation in H1299 cells; and Table S1, Primers used to generate HAdV-C5 mutants.
