# Supplementary material for: Protein–Protein Interactions Facilitate E4orf6-Dependent Regulation of E1B-55K SUMOylation in HAdV-C5 Infection
Source: Viruses. 2022 Feb 24;14(3):463. doi: 10.3390/v14030463 (PMC8953357; doi:10.3390/v14030463)
Supplement: Supplementary file 1 [file viruses-14-00463-s001.zip › viruses-1571390-supplementary.pdf]

viruses

Supplementary Materials for:

Protein-Protein Interactions Facilitate E4orf6-Dependent Regulation of E1B-55K SUMOylation in HAdV-C5 Infection

by M. Fiedler *et al.*, 2022

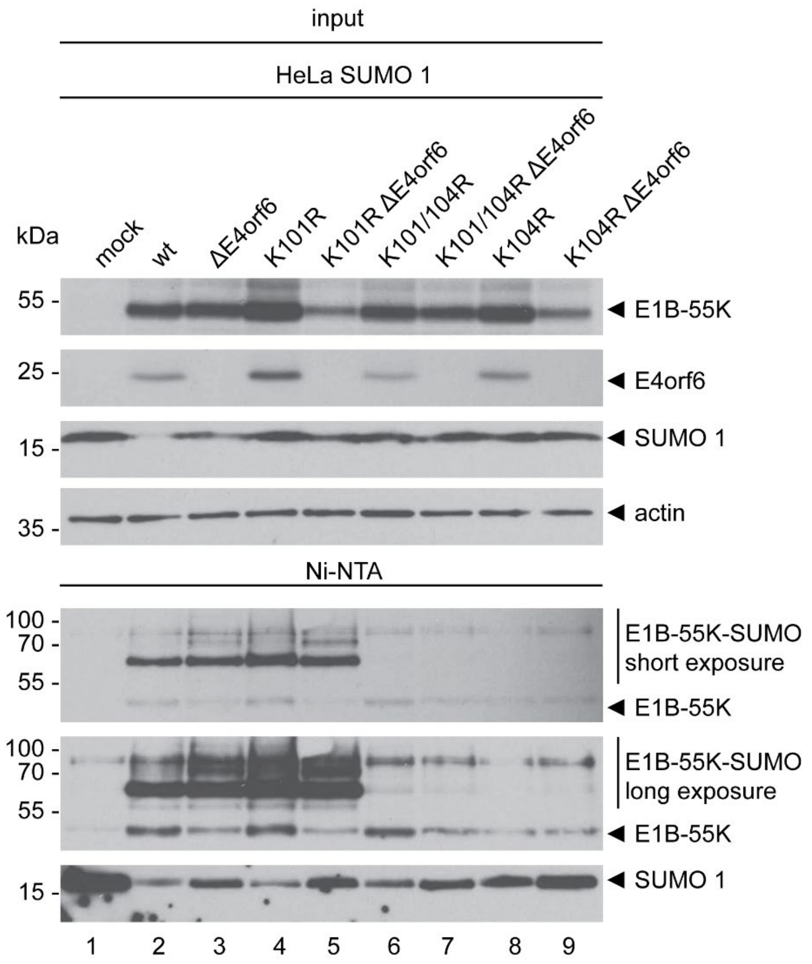

Figure S1: E4orf6 regulates E1B-55K SUMOylation and inhibits SUMO attachment. HeLa cells that constitutively express His-tagged SUMO 1 were mock infected or infected with HAdV-C5 wt and indicated mutants at an MOI of 20. Cells were harvested at 24 h p.i. and His-SUMO conjugates were Ni-NTA purified. Precipitates and total cell lysates were separated according to their molecular weight by SDS-PAGE and visualized by Western blotting. For specific protein detection, mAb 2A6 (E1B-55K), mAb RSA3 (E4orf6), mAb 6×His (SUMO 1/2) and mAb AC-15 (actin) were used. Molecular weights in kDa are indicated on the left, while corresponding proteins are labeled on the right. Image represents the results of >3 repeated experiments.

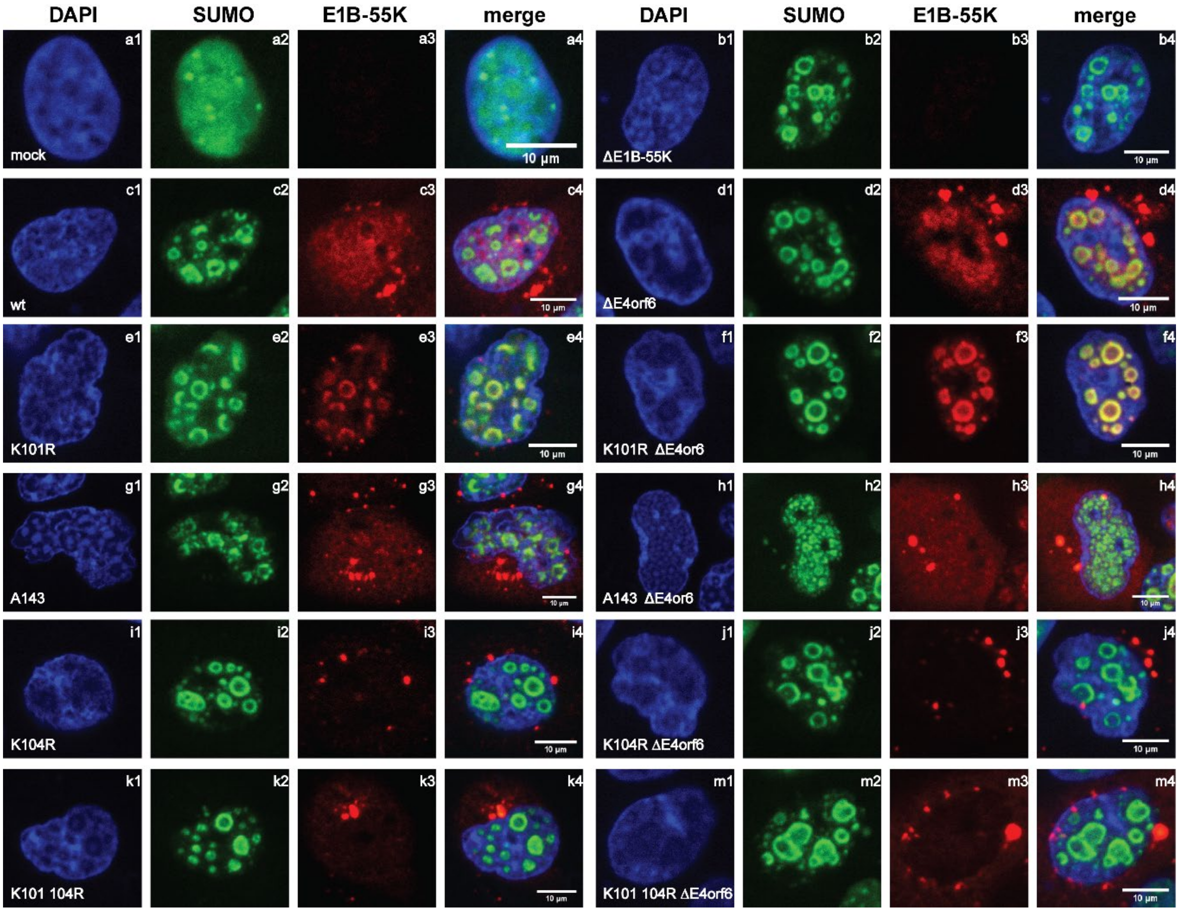

Figure S2: E4orf6 modulates the co-localization of E1B-55K and SUMO2 in viral RCs. A549 cells were either mock infected or infected with the indicated virus mutants at an MOI of 20. At 24 h p.i. cells were fixed with 4% PFA, double stained with mAb rat 4E8 (E1B-55K) and mAb mouse M114-3 (SUMO 2/3) which were detected with pAb α-rat Alexa555 (red) and pAb α-mouse Alexa488 (green). Cell nuclei were labeled with DAPI (blue). The pictures show the representative phenotypes of E1B-55K and SUMO 2, either showing no co-localization of both proteins or depicting cells in which E1B-55K- and SUMO-specific signals overlay.

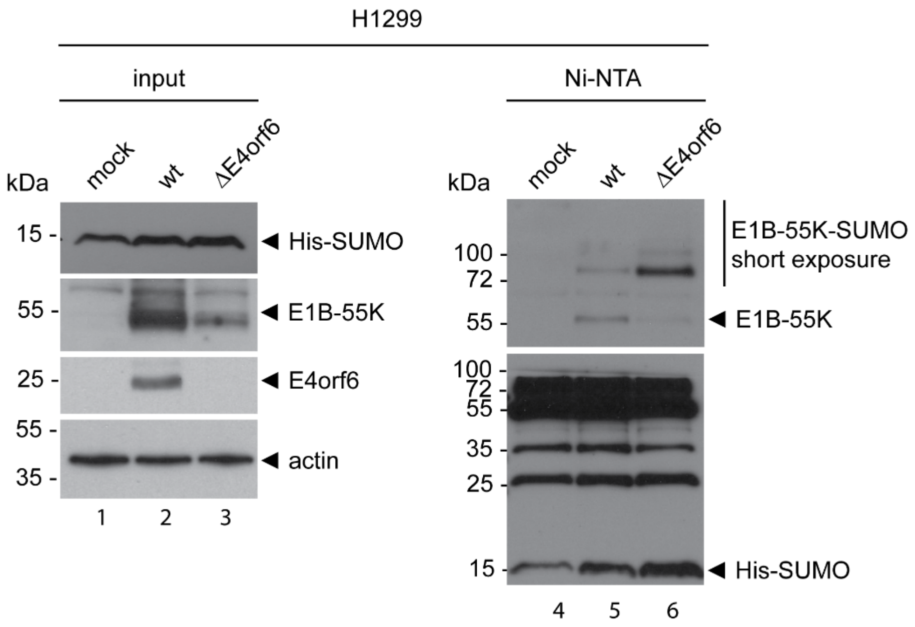

Figure S3: Validation of the E4orf6-dependent E1B-55K SUMOylation in H1299 cells. Cells were mock infected or infected at an MOI of 20 with HAdV-C5 wt or HAdV-C5 ΔE4orf6. The cells were harvested at 24 h p.i. and His-SUMO 2 conjugates were subjected to Ni-NTA purification. Total cell lysates were prepared in parallel (input). Proteins were separated by SDS-PAGE according to their molecular weight and subjected to Western blotting. Proteins were detected using mAb 2A6 (E1B-55K), mAb RSA3 (E4orf6), mAb 6×His (SUMO 1/2) and mAb AC-15 (actin). Molecular weights in kDa are indicated on the left, while corresponding proteins are labeled on the right.

Table S1: Primers used to generate HAdV-C5 mutants.

Primer		Sequence (5′ – 3′)
E1B ccdB amp	forward	CCAGAACTGAGACGCATTTTGACAATTACAGAGGATGGGCAGGGGCTA gtttgtttatttttctaaatac
	reverse	AGCTAGATTCCTAGCCTCCTCTGTAGCCTCACAAGCCCCCCGCTCCCT tttgttcaaaaaaaaagcccgctc
K101R	rescue	ATCCAGAACTGAGACGCATTTTGACAATTACAGAGGATGGGCAGGGGCTAAGGGGGGTAAAGAGGGAGCGGGGGGCTTGTGAGGCTACAGAGGAGGCTAGGAATCTAGCTTTTG
K104R	rescue	ATCCAGAACTGAGACGCATTTTGACAATTACAGAGGATGGGCAGGGGCTAAAGGGGGTAAGGAGGGAGCGGGGGGCTTGTGAGGCTACAGAGGAGGCTAGGAATCTAGCTTTT
K101/104R	rescue	ATCCAGAACTGAGACGCATTTTGACAATTACAGAGGATGGGCAGGGGCTAAGGGGGGTAAGGAGGGAGCGGGGGGCTTGTGAGGCTACAGAGGAGGCTAGGAATCTAGCTTTT
E4orf6 ccdB amp	forward	ATCCCAGGGAACAACCCATTCCTGAATCAGCGTAAATCCCACACTGCA gtttgtttatttttctaaatac
	reverse	AATGTAACACTTTGACAATGCACAACGTGAGTTACGTGCGAGGTCTTC tttgttcaaaaaaaaagcccgctc
ΔE4orf6	rescue	CATATCCCAGGGAACAACCCATTCCTGAATCAGCGTAAATCCCACACTGCAGGAAGACCTCGCACGTAACTCACGTTGTGCATTGTCAAAGTGTTACATTCGGGCA
E1B phospho ccdB amp	forward	CCAGAACTGAGACGCATTTTGACAATTACAGAGGATGGGCAGGGGCTA gtttgtttatttttctaaatac
	reverse	AGCTAGATTCCTAGCCTCCTCTGTAGCCTCACAAGCCCCCCGCTCCCT tttgttcaaaaaaaaagcccgctc
PM	rescue	AGCTGAGGCCCGATCACTTGGTGCTGGCCTGCACCCGCGCTGAGTTTGGCGATGACGATGAAGATGACGATTGAGGTACTGAAATGTGTGGGCGTGGCTTAAGGGTGGGAAAGAATAT
ΔP	rescue	AGCTGAGGCCCGATCACTTGGTGCTGGCCTGCACCCGCGCTGAGTTTGGCGCTGCCGATGAAGATGCAGATTGAGGTACTGAAATGTGTGGGCGTGGCTTAAGGGTGGGAAAGAATAT
E1B A143 ccdB amp	forward	GACACCGTCCTGAGTGTATTACTTTTCAACAGATCAAGGATAATTGCGCT gccagtatacactccgctag
	reverse	GTGGTCAGCTGCTCTATGGAATACTTCTGCGCCAGCAGATCAAGCTCATT cagccccatacgatataagttg
A143	rescue	GACACCGTCCTGAGTGTATTACTTTTCAACAGATCAAGGATAATTGCGCTCTGGAATTCCAGAATGAGCTTGATCTGCTGGCGCAGAAGTATTCCATAGAGCAGCTGACCAC

Underlined sequences indicate inserted mutations, ccdB amp specific sequences are written in lower case.
